# Mycotoxin Occurrence in Maize Silage—A Neglected Risk for Bovine Gut Health?

**DOI:** 10.3390/toxins11100577

**Published:** 2019-10-04

**Authors:** Nicole Reisinger, Sonja Schürer-Waldheim, Elisabeth Mayer, Sandra Debevere, Gunther Antonissen, Michael Sulyok, Veronika Nagl

**Affiliations:** 1 BIOMIN Research Center, Technopark 1, 3430 Tulln, Austria; nicole.reisinger@biomin.net (N.R.); sonja.schuerer@outlook.com (S.S.-W.); e.mayer@biomin.net (E.M.); 2 Department of Pharmacology, Toxicology and Biochemistry, Faculty of Veterinary Medicine, Ghent University, Salisburylaan 133, 9820 Merelbeke, Belgium; sandra.debevere@UGent.be (S.D.); gunther.antonissen@UGent.be (G.A.); 3 Department of Animal Sciences and Aquatic Ecology, Faculty of Bioscience Engineering, Ghent University, Coupure Links 653, 9000 Ghent, Belgium; 4 Department of Pathology, Bacteriology and Avian Diseases, Faculty of Veterinary Medicine, Ghent University, Salisburylaan 133, 9820 Merelbeke, Belgium; 5 Institute for Bioanalytics and Agro-Metabolomics, University of Natural Resources and Life Sciences, Vienna (BOKU), Konrad Lorenz-Straße 20, 3430 Tulln, Austria; michael.sulyok@boku.ac.at

**Keywords:** modified mycotoxin, co-occurrence, corn silage, CIEB, WST-1, NR, SRB, sphingolipid metabolism, Sa/So

## Abstract

Forages are important components of dairy cattle rations but might harbor a plethora of mycotoxins. Ruminants are considered to be less susceptible to the adverse health effects of mycotoxins, mainly because the ruminal microflora degrades certain mycotoxins. Yet, impairment of the ruminal degradation capacity or high ruminal stability of toxins can entail that the intestinal epithelium is exposed to significant mycotoxin amounts. The aims of our study were to assess (i) the mycotoxin occurrence in maize silage and (ii) the cytotoxicity of relevant mycotoxins on bovine intestinal cells. In total, 158 maize silage samples were collected from European dairy cattle farms. LC-MS/MS-based analysis of 61 mycotoxins revealed the presence of emerging mycotoxins (e.g., emodin, culmorin, enniatin B1, enniatin B, and beauvericin) in more than 70% of samples. Among the regulated mycotoxins, deoxynivalenol and zearalenone were most frequently detected (67.7%). Overall, 87% of maize silages contained more than five mycotoxins. Using an in vitro model with calf small intestinal epithelial cells B, the cytotoxicity of deoxynivalenol, nivalenol, fumonisin B1 and enniatin B was evaluated (0–200 µM). Absolute IC50 values varied in dependence of employed assay and were 1.2–3.6 µM, 0.8–1.0 µM, 8.6–18.3 µM, and 4.0–6.7 µM for deoxynivalenol, nivalenol, fumonisin B1, and enniatin B, respectively. Results highlight the potential relevance of mycotoxins for bovine gut health, a previously neglected target in ruminants.

## 1. Introduction

Mycotoxins are toxic secondary metabolites of different molds, such as *Aspergillus* spp., *Fusarium* spp., *Penicillium* spp. or *Alternaria* spp., and often found in animal feeds. They impair animal health by manifold modes of action, causing hepatotoxic, nephrotoxic, immunomodulatory, genotoxic, and neurotoxic effects as well as reproductive and developmental disorders [1]. During the last decade, the intestine has moved into the spotlight of mycotoxin research. It represents the first barrier to these feed contaminants and is often exposed to higher mycotoxin concentrations than other body tissues. Here, mycotoxins do not only affect digestion and nutrient uptake, but also intestinal histomorphology, gut barrier integrity, mucin production, microbiota composition, and the local immune system [2,3].

Due to their frequent occurrence and negative impact on animal health, many countries have established regulations for mycotoxins in feed. In the European Union (EU), maximum limits are in place for aflatoxin B1 (AFB1) and ergot alkaloids [4], while guidance levels have been set for deoxynivalenol (DON), zearalenone (ZEN), ochratoxin A (OTA) and the sum of fumonisin B1 (FB1) and fumonsin B2 (FB2) [5]. These regulations neither take the presence of multiple mycotoxins into account, nor the occurrence of so-called emerging mycotoxins. This heterogenous group of fungal metabolites is not clearly defined, but commonly referred to as “mycotoxins, which are neither routinely determined, nor legislatively regulated; however, the evidence of their incidence is rapidly increasing” [6]. Proper risk assessment of emerging mycotoxins, e.g., enniatins, culmorins, beauvericin, moniliformin, roquefortine C or fusaric acid, is challenging, as data on toxicity and occurrence are still scarce [7]. 

Forages are especially prone to contamination by emerging mycotoxins [8,9]. Fresh, dried and ensiled forages are important components of ruminant diets, representing 50–75% of the total diet [10]. Ensiling describes the preservation of green forage by lactic fermentation under anaerobic conditions and shows geographic variations concerning the quantity and type of silage produced [11]. In the EU-28 alone, approximately 2.4 million tons of green maize, which is mainly grown for silage, were harvested in 2018 [12]. Silages can contain a wide range of mycotoxins, that originate either from pre-harvest contamination, or from spoilage with (acid-tolerant and micro-aerobe) toxigenic fungi during storage [8]. Hence, ruminants might be exposed to a plethora of mycotoxins, in particular compared to chicken or swine, which have less diverse diets [9]. However, this risk has been poorly addressed so far, and the need for thorough mycotoxin monitoring in ruminant forages has been highlighted only recently [9].

In general, ruminants are considered to be less susceptible to mycotoxins than other livestock species, mainly because their ruminal microflora is capable of degrading certain mycotoxins to less toxic metabolites [8]. Most prominently, DON is extensively metabolized to de-epoxy-deoxynivalenol (DOM-1), reaching conversion rates of up to 81–99% in dairy cattle [13,14]. The close connection between a functional ruminal microflora and DON toxicity was impressively depicted by Valgaeren et al. [15]. Driven by clinical cases of DON toxicosis in 2- to 3-month-old beef calves, the authors showed that the oral bioavailability of DON is markedly increased in non-ruminating calves (50.7%) compared to ruminating calves (4.1%). Moreover, it was recently demonstrated that a low ruminal pH-value can impair the degradation of DON, NIV, ZEN, and enniatin B (ENNB) in vitro [16]. Especially in the light of subacute rumen acidosis, one of the most important nutritional diseases in dairy cattle [17], these findings are of significant practical relevance. In addition, certain mycotoxins, e.g., ENNB [18], exert antimicrobial activity. It has therefore been suggested that such mycotoxins might alter the ruminal microflora and its degradation capacity [8]. Finally, some mycotoxins hardly undergo ruminal metabolism [8]. For example, limited degradation of 10–18% was reported for FB1 [19,20]. Hence, major amounts of mycotoxins might reach the small intestine and affect gut health both in non-ruminating calves and dairy cattle.

The aims of our study were twofold. First, we investigated the mycotoxin exposure of dairy cattle. To this end, a total of 158 maize silage samples were collected in Europe and analyzed for 61 mycotoxins, including regulated as well as emerging mycotoxins. Second, an in vitro model using calf small intestinal epithelial cells B (CIEB) was established to assess the cytotoxicity of DON, NIV, FB1 and ENNB. Thus, our study does not only deliver comprehensive mycotoxin occurrence data, but also new toxicological information regarding the relevance of mycotoxins for bovine gut health, a previously neglected target in ruminants. 

## 2. Results

### 2.1. Mycotoxin Occurrence in Maize Silage 

Maize silage samples (n = 158) were collected during a 5-year period (2014–2018) at European dairy cattle farms and were analyzed for mycotoxin occurrence with a liquid chromatography–tandem mass spectrometry (LC-MS/MS)-based multi-mycotoxin method. Table 1 gives an overview on proportions of positive samples and detected mycotoxin concentrations in fresh silages.

Only two out of 158 samples (1.2%) showed no mycotoxin contamination (all mycotoxins < limit of detection). The presence of regulated mycotoxins was absent or marginal in the case of aflatoxin B1 and ochratoxin A. Similarly, ergot alkaloids were only found in 2.5% of samples, with no dominant pattern on co-occurrence of individual alkaloids. However, since concentrations of ergot alkaloids were rather low, and epimerization is promoted using acidic extraction solvents, those results should not be over interpreted. In comparison, the Fusarium toxins ZEN and DON showed a high prevalence of 67.7% each. Among the regulated mycotoxins, the highest median and maximum values were obtained for DON with 303 µg/kg and 3060 µg/kg, respectively. None of the samples exceeded the EU maximum/guidance levels set for aflatoxins, DON, FB1+FB2, ochratoxin A, or ergot alkaloids [4,5]. For the latter, it should be noted that the respective EU directive refers to the content of rye ergot (*Claviceps purpurea*; 1000 mg/kg), whereas concentrations of individual ergot alkaloids were determined in our study. Values of the 14 ergot alkaloids were adjusted to a dry matter content of 88% and summed up, yielding a maximum of 103 µg/kg total ergot alkaloids found in a silage sample from Germany. In contrast, eight samples (5.1%) contained ZEN levels ≥ 2000 µg/kg, which represents the EU guidance value for cereals and cereal products, including forages. Those samples originated from two different countries (Austria, the Netherlands) in two consecutive years (2014, 2015).

Besides DON, the highest prevalence among trichothecenes was found for NIV (59.5%) and HT-2 toxin (21.5%). Despite moderate median values, maximum NIV levels reached 5770 µg/kg in a maize silage sample from Denmark (collected in 2015). Notably, another sample from the Netherlands contained 2260 µg/kg NIV (2018), implying that prominent NIV levels were not limited to one country or season. Indicative levels for T-2+HT-2 toxin in feed were not exceeded [21]. Interestingly, 3-acetyldeoxynivalenol was not found in any of the samples, whereas the median value of 15-acetyldeoxynivaleol (274 µg/kg) was similar to the one obtained for DON. 

Furthermore, maize silages were analyzed for several modified mycotoxins, including deoxynivalenol-3-glucoside (DON-3-Glc), HT-2-toxin-3-glucoside (HT2–3-Glc) and nivalenol-3-glucoside (NIV-3-Glc). Concentrations of the modified mycotoxins did not exceed the levels of the respective parent toxins. DON-3-Glc was found in 25.3 % of samples, albeit at low levels and with an average molar percentage of D3G/DON of 2.7% (0.3–9.3%). Molar percentage for HT2–3-Glc/HT-2 toxin and NIV-3-Glc/NIV were 10.9% and 1.3% (0.9–1.6%), respectively. 

Overall, the five most frequently detected mycotoxins all belonged to the group of emerging mycotoxins: emodin (EMO) was found in 82.9% of samples, followed by culmorin (CUL; 79.1%), enniatin B1 (ENNB1; 78.5%), enniatin B (ENNB; 76.6%), and beauvericin (BEA; 76.0%). In addition, members of the emerging mycotoxins showed the highest median (5-hydroxyculmorin, 571 µg/kg) and maximum values (kojic acid 25,930 µg/kg) observed in our survey. Only six of the analyzed emerging mycotoxins were present in less than 10% of the samples, namely mycophenolic acid, enniatin B2, roquefortine C, fusaproliferin, sterigmatocystin, and enniatin B3.

**Figure 1:**
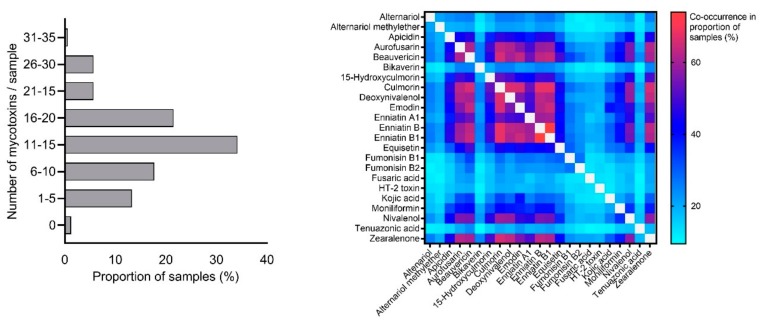
Mycotoxin co-occurrence in maize silage samples collected at European dairy cattle farms. Left: Number of mycotoxins detected per sample. Right: Prevalence of different mycotoxin combinations (only mycotoxins with individual prevalence of ≥ 20% were used for calculations).

Finally, we evaluated the co-occurrence of mycotoxins in maize silages. On average, 13 mycotoxins per sample were found (range: 0–32), and 87% of samples contained more than five mycotoxins (Figure 1, left). For assessment of the most frequently co-occurring mycotoxin combinations, toxins with an individual prevalence of ≥ 20% were considered. The most prevalent combinations were ENNB & ENNB1 (in 74.1% of samples), CUL & ENNB (67.7%), CUL & ENNB1 (67.7%), CUL & DON (66.5%), and CUL & BEA (65.8%). Figure 1 (right) illustrates all mycotoxin combinations analyzed. 

### 2.2. Cytotoxicity of Mycotoxins on Calf Small Intestinal Epithelial Cells 

**Figure 2:**
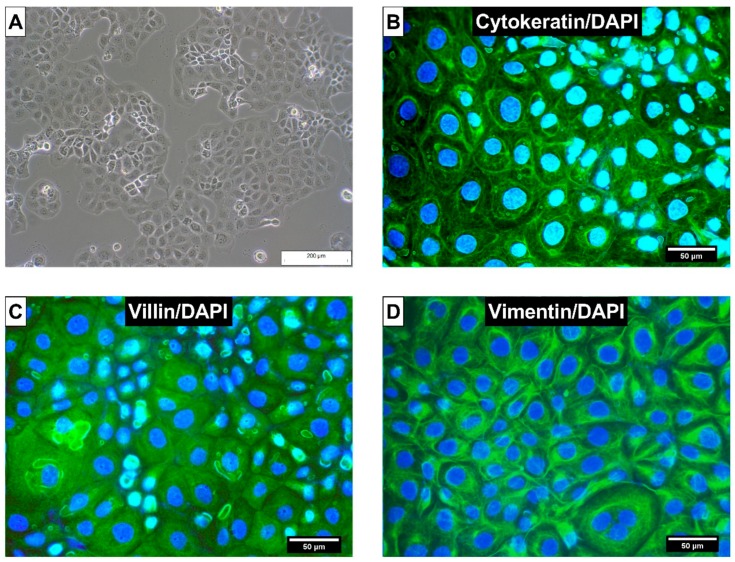
(**A**) Morphology of calf small intestinal epithelial cells B (CIEB) visualized with inverse light microscopy (passage 10, 100 × magnification). Immunostaining of CIEB in chamber slides with (**B**) cytokeratin as an epithelial cell marker, (**C**) villin as marker for intestinal cells, and (**D**) vimentin as mesenchymal marker. 4′,6-Diamidin-2-phenylindol (DAPI) was used as cell nuclei counterstain (400× magnification).

First, the species origin of used CIEB was verified via DNA Barcoding. In addition, the absence of mycoplasma contamination was confirmed prior to and throughout the experimental period. CIEB formed a cell monolayer and showed typical epithelial, cobblestone morphology (Figure 2A). For further characterization, immunohistochemistry was employed. Cytokeratins were expressed as a network radiating from the nucleus to the plasma membrane (Figure 2B), whereas villin was uniformly distributed in the cytoplasma of CIEB (Figure 2C). Vimentin was strongly expressed, forming a filamentous network throughout the cytoplasm with increased density around the nucleus (Figure 2D). As expected, isotype control antibody (Mouse IgG1,) did not show a positive reaction (Supplementary Figure S1).

The cytotoxicity of DON, NIV, FB1, and ENNB on CIEB was evaluated based on metabolic activity (WST-1 assay), lysosomal activity (NR assay), and total protein content (SRB assay). To this end, cells were treated for 48 h with increasing toxin concentrations (0–200 µM). While DON, NIV and FB1 were dissolved in culture medium, DMSO had to be used in case of ENNB due to its lower solubility. Since the DMSO proportions in the three highest ENNB concentrations (0.33–1.33% DMSO for 50–200 µM ENNB) affected the lysosomal activity of CIEB (Supplementary Table S1), respective data were excluded for calculations of IC50 values.

**Figure 3:**
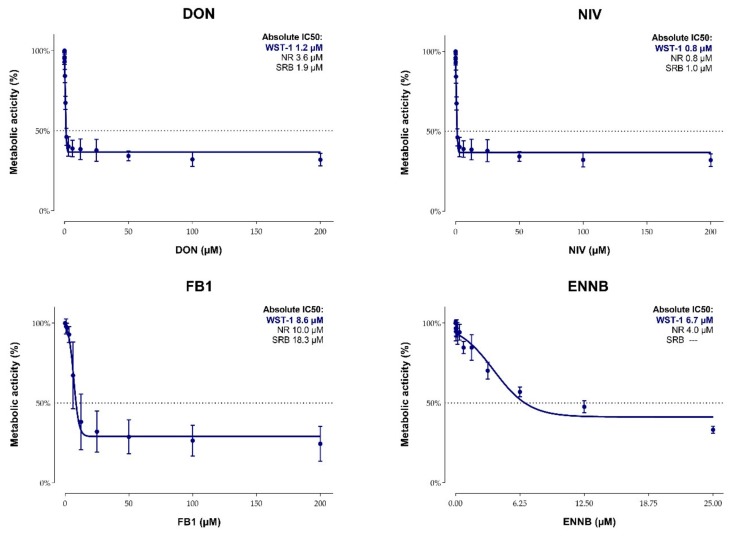
Impact of deoxynivalenol (DON), nivalenol (NIV), fumonisin B1 (FB1), and enniatin B (ENNB) on metabolic activity (%) of calf small intestinal epithelial cells B assessed via the WST-1 assay (48 h incubation, six independent experiments, three replicates per experiment). For comparison, absolute IC50 values for all three assays (WST-1, NR, SRB) are listed.

All mycotoxins tested had a dose-dependent effect on metabolic and lysosomal activity as well as on total protein content of CIEB. Obtained absolute IC50 values varied depending on the mycotoxin and assay (Figure 3). Still, some general patterns were observed. First, in all assays employed, NIV showed the highest cytotoxicity with IC50 values ranging between 0.8 and 1.0 µM, followed by DON (IC50 values 1.2–3.6 µM) and ENNB (IC50 values 4.0–6.7 µM). In comparison, FB1 showed less pronounced cytotoxic effects (IC50 values 8.6–18.3 µM). Second, the WST-1 assay showed the highest sensitivity for all tested mycotoxins except for ENNB. Here, the lowest IC50 value was obtained with the NR assay. Calculation of the absolute IC50 value for the SRB assay was not possible because the protein content never deceeded 50% in ENNB-treated cells. 

To monitor alterations of the sphingolipid metabolism in FB1-treated cells, sphinganine (Sa) and sphingosine (So) were determined in cell supernatants via LC-MS/MS. Sa was significantly increased from 25 µM FB1 onwards, whereas no influence on So concentrations was observed (Table 2). Compared to the control, a significant elevation of the Sa/So ratio was evident at 6.25–200 µM FB1. From 12.5 µM FB1 onwards, the numerical increase of the Sa/So ratio was less distinct, indicating a plateau in the response.

## 3. Discussion

Mycotoxin occurrence is influenced by multiple factors, including plant species and variety, region, temperature, humidity, insect damage, storage conditions, and other agricultural practices [22]. Our survey focused on the presence of mycotoxins in maize silage, because this feed component can be the main source for dietary mycotoxin intake in dairy cattle [23]. Since sample numbers per country and/or year were limited in our survey, definite conclusions on regional or yearly trends of mycotoxin occurrence were not justified and therefore omitted. Respective information can be retrieved from other excellent feed surveys [24] and reviews [9,11]. Similar to the approach of Storm et al. [25], mycotoxin concentrations were expressed as µg/kg fresh weight except for the comparison with EU maximum/levels, for which levels were normalized to a dry matter content of 88%. Because literature reports do not uniformly express mycotoxin concentrations in silage (using either fresh or dried weight), the suffix “fresh weight” is used in the following whenever clearly indicated in the respective study, or when samples were not dried prior to analysis.

In 98.8% of silage samples at least one mycotoxin was detected. The top five positions in terms of prevalence were all held by emerging mycotoxins, namely EMO, CUL, ENNB1, ENNB, and BEA. Although data on the presence of emerging mycotoxins in feed are scarce, high incidences of enniatins and BEA have been described previously. For example, ENNB1, ENNB, and BEA were found in 97%, 90%, and 100% of maize silages collected in Poland, respectively [26]. Reported median values (6.0–20.9 µg/kg fresh weight) were in a similar range in our study. In silage samples from Spain [27], ENNB showed yearly variations in prevalence (31–72%) with higher average concentrations (151–163 µg/kg). In contrast, moderate incidences of around 25% for ENNB [25] and BEA [28] were reported in Denmark. Differences between studies might stem from distinct fungal contamination patterns and/or variations in methodology (e.g., sampling procedure, limits of detection). Reports on the toxicity of these *Fusarium* toxins in ruminants are completely lacking so far [29]. In this respect, the described antimicrobial activity of enniatins and BEA, potentially affecting the composition and function of the rumen microbiota, might be of special interest. In addition, ENNB and BEA were demonstrated to impair the barrier function in intestinal porcine enterocytes (IPEC-J2; [30]). Since a certain proportion of ENNB might by-pass the rumen [16], negative effects on the bovine gut cannot be excluded. 

To the best of our knowledge, the occurrence of EMO and CUL in European maize silages has not been addressed yet. In line with our data, a survey conducted in Israel showed high prevalence of EMO in maize silages (100%; [31]). For CUL, results deviate from our study, mostly in terms of incidence (6.6% versus 79.1%) but also concerning median values obtained (46 µg/kg fresh weight versus 190 µg/kg). This mycotoxin has recently caught scientific attention because of its potency to inhibit DON glucuronidation [32], and we confirmed the commonly observed co-occurrence of CUL and DON [7] for maize silages. Still, the relevance of CUL for dairy cattle remains debatable, as metabolization to DOM-1 is the primary detoxification pathway for DON in ruminants. Another emerging mycotoxin that has gained certain interest is fusaric acid. Shimshoni et al. [31], authors of the aforementioned survey in Israel, pointed out both its high prevalence and concentration in maize silage. Evaluated on a larger sample size and in a different region, our findings corroborate a certain relevance for fusaric acid (detected in nearly one quarter of silages, maximum concentration of 4,120 µg/kg fresh weight). Concerns for bovine health were related to the growth inhibition of important rumen microorganisms and the toxin’s potential carry over to milk [31]. However, like for other emerging mycotoxins, toxicodynamic and toxicokinetic studies are warranted to verify these assumptions and to elucidate the role of fusaric acid for food safety. 

FB1+FB2 were detected in approximately one third of the samples, albeit at low concentrations. As unveiled by Latorre et al. [33], the majority of fumonisins in maize silage are present in a modified form. These so-called “hidden fumonisins” escape routine analysis, but are expected to be released upon mammalian digestion [34]. For assessing the total fumonisin burden, alkaline hydrolysis of samples is required [33]. This was not performed in the present case and thus represents a limitation of our study. Similarly, total exposure to type-A or -B trichothecenes is underestimated in surveys that do not account for acetylated or modified forms. In our study, DON showed a high prevalence of 67.7% with moderate median concentrations of 303 µg/kg fresh weight. In the past, higher average DON values of 1,629 µg/kg fresh weight [25] or 854–1316 µg/kg [10,27] were monitored, and incidences varied substantially from 6.1–86% [25,26,27,35]. While average molar DON-3-Glc/DON percentages of 20% were proposed for cereals [36], we found markedly lower values of 2.72%. Further studies are necessary to assess whether this observation is related to the commodity maize silage as such or merely to our sample set. The same applies to our findings on NIV-3-Glc and HT2–3-Glc, both showing negligible prevalence. Although this indicates that NIV-3-Glc does not contribute significantly to the total NIV burden of dairy cattle, the prominent prevalence of the parent toxin (59.5%) must be underlined. Maximum NIV values exceeded previously reported data [25,26,37], revealing that the NIV exposure can be extremely high for individual dairy cattle herds. 

ZEN was the only mycotoxin found at levels above the EU maximum/guidance limits [4,5], with 5.1% of samples exceeding ≥ 2,000 µg/kg ZEN. In most mycotoxin surveys, maize silages complied to the EU regulations [10,25,26,37], whereas Dangac et al. [27] reported 1.4% of samples exceeding the recommended maximum levels for ZEN. It should be noted that a different limit was employed in that study (500 µg/kg ZEN for complete feedstuffs), which hampers a direct comparison of results. Still, data emphasize the need to monitor ZEN in maize silage and to control its formation pre- and postharvest. This is especially important in the light of potential synergistic effects with other mycotoxins. Naturally, ZEN often co-occurred with DON (63.3%). While exposure to diets co-contaminated with ZEN and DON did not affect the performance of dairy cows [38], alterations of health-related blood parameters were observed by Dänicke et al. [39]. In addition, authors suggested an influence on ketogenesis at the cellular level. Clearly, more studies are needed to decipher the interactions of DON and ZEN in ruminants. The same is valid for other mycotoxin combinations.

Co-occurrence of mycotoxins might be of relevance for animal health even at comparably low concentrations. As summarized by Chehli et al. [40], the type and intensity of mycotoxin interactions can vary dose-dependently. Our study confirmed that mycotoxin co-occurrence in feed is rather the rule than the exception. Strikingly, silage samples contained 13 mycotoxins on average, and in 87% of samples more than five mycotoxins were found. These high values are partly attributed to the broad palette of mycotoxins tested in our study, and therefore, further expand existing knowledge on mycotoxin co-contamination in maize silage (e.g., Refs. [9,26,27]). 

Next, we investigated the impact of silage mycotoxins on bovine gut health. Based on our survey results, we focused on *Fusarium* toxins and assessed the cytotoxic potential of DON, NIV, FB1, and ENNB on bovine intestinal cells. Toxins were selected due to their high prevalence (DON, ENNB), maximum concentrations (NIV) or ruminal stability even under physiological conditions (FB1; [20]). The toxicity of mycotoxins in bovine intestinal cells is currently unknown, mainly because of two reasons. First, the rumen microbiota was long thought to neutralize the toxicity of mycotoxins. However, recent studies indicate that the ruminal degradation capacity might be impaired under specific conditions, such as altered ruminal development in calves [15] or rumen acidosis [16]. Second, the small number of commercially available bovine lines restrains research in this field.

Since CIEB are not widely used, we first confirmed the species identity and the absence of mycoplasma contamination. Although these aspects are of paramount importance for reliable and reproducible in vitro results, they are often neglected. Mycoplasma contamination can alter the properties of cell lines, and infected CIEB were described to exhibit low viability and poor growth [41]. In the same study, authors reported misidentification of three out of eight tested cell lines. The dimension of this issue is even more striking when retrieving information from the International Cell Line Authentication Committee, which has documented 451 false identified cell lines [42]. Resources wasted in the last 50 years due to misidentification of cell lines, stemming from cross-contamination, wrongly labelled samples or inadequate protocols, can only be estimated [43]. Consequently, increased attention should be paid to adequate quality controls for in vitro experiments, also in mycotoxin research.

Further characterization of CIEB was performed by immunofluorescence staining. CIEB showed a positive reaction for cytokeratins and villin. Cytokeratin proteins, which are characteristic components of the cytoskeleton, are commonly used for identification of epithelial cells [44]. Villin is an actin-binding protein in the microvilli of epithelial cell [45]. Expression of both proteins has been used to verify the intestinal epithelial nature of bovine cells before [46]. Besides, CIEB were immuno-positive for vimentin. This protein is a typical marker for non-epithelial cells, such as fibroblasts [47]. However, unequivocal identification of fibroblasts remains challenging. For example, the expression of vimentin was reported for the intestinal porcine epithelial cell line (IPEC-J2; [48]). Another study even excluded the presence of fibroblasts in cells isolated from calf intestine although they showed a positive reaction for vimentin [49]. It seems that the expression of vimentin is not a unique property of mesenchymal cells but can also be found in intestinal epithelial cells and should be evaluated in combination with the presence/absence of cytokeratin expression. Altogether, our immunohistochemistry results asserted the epithelial intestinal origin of CIEBs.

Absolute IC50 values of mycotoxins were calculated based on viability tests performed with three different assays (WST-1, SRB, NR). Independent of the assay, NIV was the most cytotoxic mycotoxin (IC50 0.8–1.0 µM), closely followed by DON (1.2–3.6 µM). The higher comparative cytotoxicity of NIV is in accordance to experiments performed in human (epithelial colorectal adenocarcinoma cells, Caco-2; [50]) and porcine intestinal cells (IPEC-1, IPEC-J2; [51,52]). Likewise, the absolute IC50 values obtained for NIV and DON in CIEB are in a similar range as reported previously. For example, IC50 values for NIV were 0.9–2.1 µM in Caco-2, IPEC-1 and IPEC-J2 cells [50,51,52], and 0.9–3.6 for DON [50,51,52,53]. Opposed to that, individual studies found higher IC50 values, e.g., 6.9 µM for NIV [54] or up to 44.8 µM for DON [55]. Differences between studies can derive from experimental conditions, such as cultivation medium, tested concentration range, exposure period, chosen endpoint, calculation of IC50 values, or differentiation status of cells [40]. Overall, data indicate that CIEB are at least as sensitive to NIV and DON as human or porcine intestinal cells.

For ENNB, a higher cytotoxicity compared to NIV [54] and DON [56] was observed in Caco-2, which could not be confirmed for CIEB. Interestingly, the most sensitive IC50 value for ENNB (4.0 µM) was generated by the NR assay, which measures lysosomal activity. Indeed, destabilization of lysosomes has been suggested as an upstream event of ENNB-induced cell death [57]. As IC50 values after incubation periods of up to 48 h varied strongly in Caco-2 (2.1 to > 30 µM; [18]), comparison of results is challenging. Still, in line with the present study, it was reported that the NR assay yields lower IC50 values for ENNB than assays measuring metabolic activity [57,58]. Since mitochondria are one of the major cellular targets of enniatins [18], we originally assumed a strong response in the WST-1 assay. However, among other effects on these cell organelles, enniatins induced swelling of rat liver mitochondria [59]. Interestingly, the same phenomenon was described for IPEC-J2 cells exposed to DON, and here the comparably weaker cytotoxic response assessed by the WST-1 assay was partly attributed to alterations of the mitochondrial morphology and metabolic activity [30]. Although further mechanistic studies are needed, cumulative data suggest that the metabolic activity does not represent the most sensitive endpoint for cytotoxicity assessment of ENNB. For the SRB assay, calculation of an IC50 value was not possible. To the best of our knowledge, no other study has employed this test to determine the cytotoxicity of ENNB so far. Hence, we cannot conclude whether the total protein content is the least sensitive endpoint for this mycotoxin or whether this finding is rather limited to our experimental conditions. Concordant with Springler et al. [55], our study underlines the importance of multi-parameter analysis in the cytotoxicity assessment of mycotoxins. 

FB1 showed the lowest cytotoxicity among the toxins tested in our study. Considering previous studies demonstrating minor cytotoxicity of this mycotoxin in Caco-2 [60,61,62,63] and IPEC-J2 [52,64], these results are not surprising. Still, it should be noted that IC50 values obtained in CIEB are markedly lower than the ones reported previously for other intestinal cells (if computable at all). This might be partly related to the narrow concentration range tested in some of the studies [52,60,63]. FB1-induced effects on the intestine were proposed to originate from disruption of the sphingolipid metabolism which causes intracellular accumulation of the sphingoid bases sphinganine (Sa) and sphingosine (So) [65]. In line with reports addressing intestinal tissues/cells from monogastric livestock species [66,67], a dose-dependent increase of the Sa/So ratio was observed in CIEB. Compared to Loiseau et al. [66], who found a significant elevation of the Sa/So ratio after 48 h of exposure to 100 µM FB1 in IPEC-J2, CIEB reacted to lower toxin concentrations, reaching statistical significance at 6.25 µM FB1. Yet, absolute Sa/So values were smaller in our study, which might be explained by the type of matrix used for analysis (cell extract [66] or supernatant). 

Although ZEN showed high prevalence in silage samples and was the only mycotoxin exceeding the EU guidance levels, we did not include this compound in our cytotoxicity experiments. This decision was mainly based on the primary mode of action of ZEN, which is the activation of estrogen receptors [68]. Compared to other mycotoxins, the effects of ZEN on the intestine are less detrimental [69]. For example, IC50 values for ZEN obtained by measuring metabolic activity in Caco-2 were 313 µM [70] and 25 µM [62] after 48 and 72 h of incubation, respectively, and thus even higher than those observed for FB1 in the same experiments. However, increased sensitivity of CIEB to ZEN cannot be ruled out at this stage and should be addressed in future studies. In vitro models represent an essential tool to unravel the toxicological relevance and mode of action of substances. Yet, direct extrapolation to in vivo conditions is often limited, mainly because in-vitro experiments cannot fully reflect the complexity of an intact organism [40]. In an attempt to compare concentrations used in our in vitro experiment to mycotoxin levels in dairy feed, we used the dataset provided by Seeling et al. [14]. In this study, 14 duodenal fistulated cows were exposed to DON-contaminated feed, which allowed the assessment of the toxin’s duodenal flow. On average, 1.3% on ingested DON reached the duodenum in unmetabolized form. This low proportion partly stemmed from ruminal absorption of the toxin, but mostly from metabolization to DOM-1 (94–99%). Calculating with this percentage, the IC50 value for DON in CIEB (356 µg/L; WST-1) theoretically corresponds to a feed concentration of approximately 27,400 µg/kg. Although this value exceeds the maximum DON levels detected in fresh maize silage by a factor of ten, it should not be overlooked that minor changes in the ruminal degradation capacity would have a marked impact on the outcome of this estimation. As such, it highlights the practical relevance of our findings. 

## 4. Conclusions

Our survey reports a high prevalence of emerging mycotoxins, namely EMO, CUL, enniatins and BEA, in European maize silages. In addition, the well-known *Fusarium* toxins ZEN, DON and NIV were frequently detected, often co-occurring with the listed emerging mycotoxins. Based on the comparison of obtained IC50 values, our data indicate that CIEB are at least as sensitive to NIV, DON, ENNB and FB1 as human or porcine intestinal cells. Thus, our study stresses the potential negative health impact of mycotoxins on bovine gut health and highlights the need for further research in this field. In particular, effects of mycotoxin combinations on the composition and functionality of the rumen microbiota as well as on bovine gut health in vivo should be addressed 

## 5. Materials and Methods 

### 5.1. Mycotoxin Survey 

**Figure 4:**
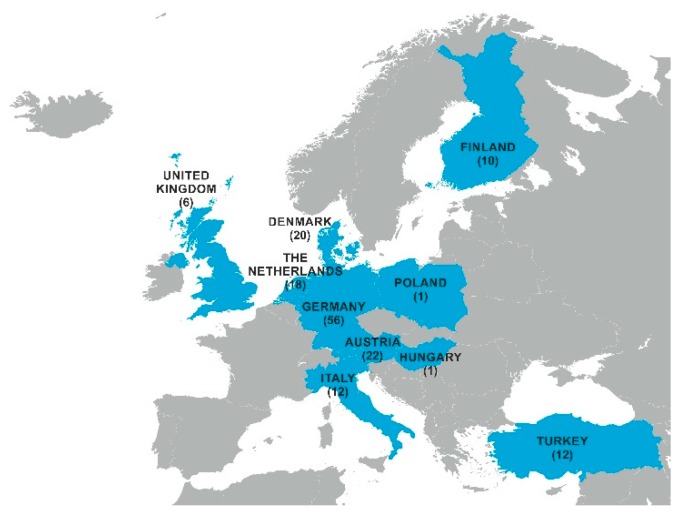
Number of maize silage samples per country of origin.

In total, 158 maize silage samples were collected at European dairy cattle farms from January 2014 to December 2018. Per year, 19 (2014), 20 (2015), 51 (2016), 36 (2017), and 31 (2018) samples were taken. The 10 countries of origin are displayed in Figure 4.

Samples were provided by the BIOMIN Mycotoxin Survey Program and collected as described previously [71]. Feed was sent for analysis in paper bags or bags with ventilation to avoid humidity building up. Prior to LC-MS/MS analysis, aliquots of 500 g were dried (60 °C, 48 h; drying cabinet FP 24, Binder GmbH, Tuttlingen, Germany), and the dry matter content was determined simultaneously. Thereafter, dried samples were homogenized, extracted and subjected to LC-MS/MS-based multi-mycotoxin analysis according to Malachovà et al. [72]. Details regarding the identification and quantification of mycotoxins as well as the method performance are reported in the aforementioned publication. The accuracy of the method is verified by regular participation in proficiency testing schemes including samples of complex animal feed [72,73]. 

Final mycotoxin concentrations were corrected for dry matter content and expressed as µg/kg fresh weight (average dry matter content 36.5 ± 8.6%). Samples with mycotoxin levels below the limit of detection (LOD) were considered negative. In case samples shown a mycotoxin concentration between the LOD and limit of quantification (LOQ), LOQ/2 was used to calculate median and percentile values. For evaluation of samples exceeding the EU legislation on maximum/guidance/indicative mycotoxin levels in feed [4,5,21], values were normalized to a dry matter content of 88%. Specifically, the guidance values for the category “Feed materials – Cereals and cereal products” were used for DON (8,000 µg/kg), ZEN (2,000 µg/kg) and ochratoxin A (250 µg/kg). In the case of FB1, the guidance level for “Feed materials – Maize and maize products” was selected (60,000 µg/kg), whereas for the sum of T-2 and HT-2 toxins, the indicative level for “Cereal products for feed and compound feed – Other cereal products” (500 µg/kg) was used. 

### 5.2. In vitro Experiments 

#### 5.2.1. Cell Line 

Calf small intestinal epithelial cells B (CIEB) are a spontaneously immortalized cell line from *bos taurus* (NCBI Taxonomy: 9913). CIEB clone 9 (RRID:CVCL_6A77) were originally purchased from Bionutritec (Iunel, France).

Prior to the conduction of experiments, cells were checked for the absence of mycoplasma contamination with the broth-agar microbiological culture method (Friis and PH broth and agar media; German Collection of Microorganisms and Cell Cultures, Braunschweig, Germany). In addition, CIEB were sent to DSMZ for confirmation of species identification. Cells were tested to be free of mitochondrial DNA sequences from human (detection limit: 10^–3^) as well as from mouse, rat, and Syrian and Chinese hamster (detection limit:10^–5^). DNA Barcoding by PCR amplification of the 5´-coding region of Cytochrome C Oxidase I and sequencing of the respective PCR product was used to confirm the species origin of CIEB. 

#### 5.2.2. Routine Cultivation of CIEB

Cells were maintained in high glucose (4.5 g/L) Gibco® D-MEM medium (Invitrogen, Thermo Scientific, Vienna, Austria). Media was supplemented with 10% fetal bovine serum (Life Technologies, Thermo Scientific, Vienna, Austria), 16 mM HEPES (Sigma Aldrich, Vienna, Austria), Gibco® 2.5 mM GlutaMAX™, and penicillin/streptomycin (100 units/mL; 100 µg/mL; Sigma Aldrich)). CIEB were cultivated at 37 °C in a 5% CO2 atmosphere (Galaxy 48 S, New Brunswick, Eppendorf, Hamburg, Austria). At first passage, coated (Coating Matrix Kit, Life Technologies) 25-flasks (Star Lab, Hamburg, Germany) were used. Thereafter, cells were cultivated in uncoated 75-flasks (Star Lab). CIEB were subcultured two to three times a week upon reaching 80% confluence. Cells were used until passage 24 and regularly confirmed to be free of mycoplasma contamination via PCR (Venor® GeM Mycoplasma Detection Kit; Minerva Biolabs, Berlin, Germany).

#### 5.2.3. Characterization of CIEB by Immunohistochemistry (Cytokeration, Vimentin and Villin)

For antibody staining, eight well cell imaging slides (Eppendorf, Hamburg, Germany) were coated with a Coating Matrix Kit (Life Technologies). CIEB were seeded at a density of 2 × 10^4^ cells/well and incubated for 48 h at 37 °C and 5% CO2. Cells were washed twice with PBS before fixing them with 4% paraformaldehyde solution for 10 minutes at 4 °C. Subsequently, cells were washed again with PBS. For permeabilization, cells were incubated with PBS containing 0.1% Triton X-100 at 200 rpm followed by another washing step with PBS. Blocking was performed by incubating cells for 1 h at 200 rpm with a 2% BSA solution at room temperature, followed by a washing step with PBS. Thereafter, cells were incubated with primary antibodies for 1 h at room temperature: mouse anti-pan cytokeratin antibody [C11] (1:250 dilution, ab7753, Abcam), mouse anti-vimentin antibody [RV202] (1:150, dilution, ab8978, Abcam), mouse anti-villin antibody [3E5G11] - N-terminal (1:250 dilution, ab201989, Abcam). Mouse IgG1, Kappa Monoclonal [B11/6]–Isotype Control (1:250 dilution, ab91353, Abcam) was used as isotype control. After another washing step, cells were incubated for 2 h at room temperature in the dark with the secondary antibody: goat anti-mouse IgG H&L FITC (1:2,000, ab6785, Abcam). Thereafter, cells were washed again, and three drops/well of a fluoroshield mounting medium containing DAPI (Abcam) were added for 5 minutes at room temperature in the dark. Finally, the chambers of the imaging slides were removed, and cover glasses were added. 

#### 5.2.4. Cytotoxicity Tests

Solid mycotoxin standards were purchased from Romer Labs (Tulln, Austria; DON, NIV and FB1) or Sigma Aldrich (ENNB). For the preparation of stock solutions, 5 mg of DON, NIV and FB1 were dissolved in appropriate amounts of culture medium to yield a concentration of 1 mM, respectively. In the case of ENNB, 5 mg of the standard were dissolved in DMSO to achieve a concentration of 15 mM. All stock solutions were stored at −20 °C. 

CIEB were seeded in 96-well plates (Eppendorf, Vienna, Austria) with a density of 2 × 10^4^ cells/well and 200 µL medium/well. After 24 h, cells were incubated with DON, NIV, FB1 (0–200 µM) or ENNB (0–200 µM; DMSO 0–1.33%) in triplicate for 48 h. Thereafter, three different assays were performed to assess the cytotoxicity of increasing mycotoxin concentrations. First, the water-soluble tetrazolium (WST-1; Roche, Rotkreuz, Switzerland) was performed to assess the metabolic activity of CIEB. While the sulforhodamine B assay (SRB; Xenometrix, Allschwil, Switzerland/Sigma Aldrich) was used to measure the total protein synthesis of cells, the neutral red assay (NR, Xenometrix) was used the measure the ability of viable cells to incorporate and bind neutral red within lysosomes. All tests were performed according to the instructions of the manufacturer. Six independent experiments were performed for each mycotoxin and test, respectively. 

#### 5.2.5. Sphinganine and Sphingosine Analysis 

In supernatants of FB1-treated cells, the concentration of the sphingoid bases sphinganine (Sa) and sphingosine (So) were determined via LC-MS/MS as described in Reisinger et al. [74]. Supernatants from triplicates were pooled, and four independent experiments were performed.

#### 5.2.6. Data Analysis 

Statistical analysis was performed with GraphPad Prism (Prism version 8 for Windows, GraphPad Software, La Jolla, California, USA). Absolute IC50 values of data from the WST-1, NR and SRB assays were calculated with relative numbers. After data normalization, a four-parameter nonlinear regression curve [log (inhibition) versus response with variable slope (least squares ordinary fit, with the condition that the Hillslope is < 0)] was applied to calculate the IC50 values. Sa/So ratios were calculated by dividing the Sa concentration by the So concentration of each experiment. As data were not normally distributed, the Kruskal-Wallis test was performed as a non-parametric test. Dunnett’s test was used as a post-hoc test to compare different FB1 concentrations against cell control (0 µM FB1).

For the extrapolation of DON concentrations used in vitro to DON levels in feed, data from Seeling et al. [14] were used. Average DON recovery in duodenum (% of ingested DON) was calculated based on the individual data provided for 14 cows in table 3 of the publication.

## Figures and Tables

**Table 1:** Occurrence of tested mycotoxins in 158 dairy maize silage samples collected in Europe from 2014 to 2018. Mycotoxin concentrations are expressed as µg/kg fresh silage. Numbers in bold indicate the top five values per category (e.g., highest number of positive samples, highest median, etc.).

Mycotoxin	Positive Samples ^1^ (n)	Positive Samples ^1^ (%)	Median Concentration ^2^ (µg/kg)	75^th^ Percentile ^2^ (µg/kg)	95^th^ Percentile^2^ (µg/kg)	Maximum Concentration (µg/kg)
*Regulated mycotoxins (except ergot alkaloids) ^3^*						
Aflatoxin B1 ^4^	0	0.0	-	-	-	-
Deoxynivalenol	107	67.7	303	556	1490	3060
Fumonisin B1	55	34.8	60.0	147	262	553
Fumonisin B2	46	29.1	20.4	34.4	101	133
Ochratoxin A	4	2.5	2.38	2.51	2.62	2.65
Zearalenone	107	67.7	15.2	61	1110	1670
*Ergot alkaloids ^3^*						
Ergine	0	0.0	-	-	-	-
Ergocornine	0	0.0	-	-	-	-
Ergocorninin	0	0.0	-	-	-	-
Ergocristine	0	0.0	-	-	-	-
Ergocristinine	0	0.0	-	-	-	-
Ergocryptine	2	1.3	5.41	7.77	9.65	10.12
Ergocryptinine	0	0.0	-	-	-	-
Ergometrine	1	0.6	49.6	49.6	49.6	49.6
Ergometrinine	1	0.6	3.20	3.20	3.20	3.20
Ergosin	2	1.3	1.89	1.90	1.91	1.91
Ergosinin	0	0.0	-	-	-	-
Ergotamine	1	0.6	1.54	1.54	1.54	1.54
Ergotaminine	0	0.0	-	-	-	-
Ergovalin	0	0.0	-	-	-	-
*Type-A trichothecenes*						
Diacetoxyscirpenol	0	0.0	-	-	-	-
HT-2 toxin	34	21.5	14.7	21.4	51.9	90.2
Monoacetoxyscirpenol	4	2.5	9.91	15.9	29.5	32.9
Neosolaniol	0	0.0	-	-	-	-
T-2 toxin	6	3.8	2.55	2.89	3.79	4.08
*Type-B trichothecenes*						
3-Acetyldeoxynivalenol	0	0.0	-	-	-	-
15-Acetyldeoxynivalenol	8	5.1	274	480	624	687
Nivalenol	94	59.5	113	237	623	5770
*Modified mycotoxins*						
Deoxynivalenol-3-glucoside	40	25.3	17.1	49.2	121	129
HT-2-toxin-3-glucoside ^5^	1	0.6	6.28	6.28	6.28	6.28
Nivalenol-3-glucoside ^5^	5	3.2	6.01	6.06	9.68	10.6
α-zearalenol	12	7.6	4.84	6.93	18.1	22.2
β-zearalenol	8	5.1	4.90	6.66	12.4	12.6
*Emerging mycotoxins*						
Alternariol	45	28.5	3.11	4.45	12.1	48.1
Alternariol methylether	37	23.4	1.95	3.46	5.79	30.8
Apicidin	79	50.0	9.49	25.0	102	175
Aurofusarin	108	68.4	97.8	307	3840	4710
Beauvericin	120	76.0	9.16	19.0	75.9	214
Bikaverin	42	26.6	20.3	58.8	248	415
Butenolid	30	19.0	28.9	70.9	249	583
Culmorin	125	79.1	190	719	2930	6680
5-Hydroxyculmorin	19	12.0	571	989	1400	1480
15-Hydroxyculmorin	84	53.2	229	504	1520	1670
15-Hydroxyculmoron	22	13.9	204	396	441	484
Emodin	131	82.9	4.38	14.1	211	1640
Enniatin A	30	19.0	2.45	5.23	32.5	50.1
Enniatin A1	98	62.0	2.70	8.73	25.2	173.9
Enniatin B	121	76.6	7.07	13.8	47.4	429
Enniatin B1	124	78.5	5.68	15.5	46.7	555
Enniatin B2	8	5.1	3.40	5.49	16.0	20.7
Enniatin B3	0	0.0	-	-	-	-
Equisetin	86	54.4	4.75	8.42	17.4	45.4
Fusaproliferin	4	2.5	170	286	316	322.3
Fusaric acid	35	22.2	229	998	1800	4120
Kojic acid	67	42.4	96.3	185	876	25,930
Moniliformin	71	44.9	7.84	18.5	61.6	113
Mycophenolic Acid	9	5.7	14.8	80	262	352
Roquefortine C	7	4.4	11.7	21.3	326	454
Sterigmatocystin	3	1.9	2.38	5.89	8.65	9.35
Tenuazonic acid	42	26.6	60.6	182	574	727

^1^ Samples with values > limit of detection (LOD); ^2^ Excluding data < LOD. In case values were between LOD and limit of quantification (LOQ), LOQ/2 was used for calculation; ^3^ According to regulations/recommendations set by the European Commission for dairy feeds [4,5]; ^4^ All samples below < LOD for aflatoxin B2, aflatoxin G1 and aflatoxin G2; ^5^ Included in analysis from 2016 onwards.

**Table 2:** Sphinganine (Sa) and sphingosine (So) concentrations as well as sphinganine to sphingosine ratio (Sa/So) in supernatants of calf small intestinal epithelial cells B treated with increasing concentrations of FB1 (0–200 µM; n = 4 independent experiments). ^a,b^ Superscripts indicate significant differences to cells incubated without FB1 (0 µM).

FB1 (µM)	Sa (ng/mL)	So (ng/mL)	Sa/So
0	0.21 ± 0.81 ^a^	1.40 ± 0.40	0.15 ± 0.02 ^a^
0.781	0.28 ± 0.10 ^a^	1.42 ± 0.33	0.20 ± 0.03 ^a^
1.563	0.53 ± 0.25 ^a^	1.70 ± 0.47	0.31 ± 0.06 ^a^
3.125	3.89 ± 1.51 ^a^	1.61 ± 0.25	2.36 ± 0.55 ^a^
6.25	15.60 ± 5.91 ^a^	1.53 ± 0.25	9.98 ± 2.20 ^b^
12.5	33.61 ± 13.29 ^a^	1.96 ± 0.64	16.96 ± 2.11 ^b^
25	44.57 ± 17.19 ^b^	2.36 ± 1.00	19.11 ± 1.71 ^b^
50	50.64 ± 25.01 ^b^	2.49 ± 1.11	20.08 ± 0.86 ^b^
100	53.57 ± 23.27 ^b^	2.29 ± 0.90	23.25 ± 1.15 ^b^
200	56.13 ± 26.26 ^b^	2.16 ± 0.83	25.58 ± 2.11 ^b^
p-value	<0.0001	0.0462	<0.0001

## Supplementary Materials

The following are available online at https://www.mdpi.com/2072-6651/11/10/577/s1, Figure S1: Immunostaining of calf small intestinal cells B (CIEB) in chamber slides with isotype control antibody for cytokeratin, villin and vimentin, Table S1: Impact of the solvent DMSO (0–10%) on metabolic activity (%) of calf small intestinal epithelial cells B assessed via the WST-1, NR, and SRB assay (48 h incubation, four independent experiments, three replicates per experiment).
