# Supplementary material for: Mycotoxin Occurrence in Maize Silage—A Neglected Risk for Bovine Gut Health?
Source: Toxins (Basel). 2019 Oct 4;11(10):577. doi: 10.3390/toxins11100577 (PMC6832361; doi:10.3390/toxins11100577)
Supplement: Supplementary file 1 [file toxins-11-00577-s001.pdf]

Supplementary material: Mycotoxin Occurrence in Maize Silage – a Neglected Risk for Bovine Gut Health?

Nicole Reisinger, Sonja Schürer-Waldheim, Elisabeth Mayer, Sandra Debevere, Gunther Antonissen, Michael Sulyok and Veronika Nagl

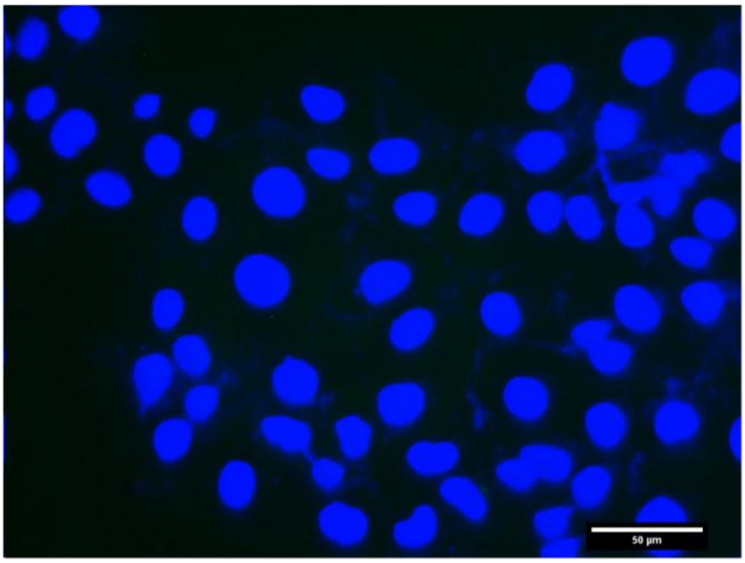

Figure S1. Immunostaining of calf small intestinal cells B (CIEB) in chamber slides with isotype control antibody for cytokeratin, villin and vimentin. 4′,6-Diamidin-2-phenylindol (DAPI) was used as cell nuclei counterstain (400× magnification). Isotype control Mouse IgG1 did not show a positive reaction.

For evaluating the effects of DMSO on cell viability of CIEB, cells were exposed to increasing concentrations of DMSO (0 – 10%; n = four independent experiments; three replicates per experiment) for 48 hours. Thereafter, metabolic activity, lysosomal activity and protein content were determined as described under 5.1.4. For statistical evaluation, the Kruskal-Wallis test was performed as non-parametric test, since data were not normally distributed. Dunnett's test was used as post hoc test to compare different DMSO concentrations against the cell control. As can be derived from Supplementary Table S1, DMSO concentrations from 0.33% onwards affected lysosomal activity.

Table S1. Impact of the solvent DMSO (0–10%) on metabolic activity (%) of calf small intestinal epithelial cells B assessed via the WST-1, NR and SRB assay (48 hours incubation, four independent experiments, three replicates per experiment). [a,b] Subscripts indicate significant difference to cell control (0% DMSO) which was set to 100%.

DMSO (%)	Metabolic Activity (%, WST-1)	Lysosomal Activity (%, NR)	Protein Content (%, SRB)
0.1	89.19 ± 1.65 [a]	99.47 ± 1.23 [a]	98.94 ± 0.90 [a]
0.16	93.90 ± 3-16 [a]	100.50 ± 1.62 [a]	93.23 ± 1.12 [a]
0.33	79.42 ± 1.56 [a]	96.69 ± 1.46 [b]	88.72 ± 2.26 [a]
0.5	74.81 ± 4.38 [a]	91.34 ± 2.16 [b]	89.48 ± 1.01 [a]
0.66	73.55 ± 4.32 [a]	94.15 ± 2.27 [b]	82.48 ± 1.16 [a]
1	60.61 ± 2.26 [b]	88.89 ± 1.47 [b]	81.78 ± 1.72 [a]
1.33	60.57 ± 3.97 [b]	93.77 ± 2.17 [b]	73.86 ± 1.71 [b]
1.5	57.99 ± 1.74 [b]	86.26 ± 2.24 [b]	76.85 ± 1.45 [b]
2	52.33 ± 2.00 [b]	85.41 ± 2.06 [b]	70.77 ± 2.11 [b]
3	53.75 ± 2.35 [b]	78.62 ± 0.83 [b]	60.46 ± 2.25 [b]
5	14.02 ± 2.52 [b]	2.76 ± 0.78 [b]	33.35 ± 2.79 [b]
10	0.37 ± 0.44 [b]	-0.13 ± 0.03 [b]	11.55 ± 1.38 [b]
p-value	< 0.0001	< 0.0001	< 0.0001
